# Identification of a novel mutation in the *OsMRP5* gene in low phytate Basmati rice mutant and development of CAPS marker for marker-assisted breeding

**DOI:** 10.3389/fpls.2024.1455219

**Published:** 2024-12-17

**Authors:** Zia-ul- Qamar, Muhammad Uzair, Amjad Hameed, Syed Adeel Zafar, Xueyong Li

**Affiliations:** ^1^ Plant Breeding & Genetics Division, Marker Assisted Breeding Group (MABG), Nuclear Institute for Agriculture and Biology (NIAB), Faisalabad, Pakistan; ^2^ State Key Laboratory of Crop Gene Resources and Breeding, Institute of Crop Science, Chinese Academy of Agricultural Sciences, Beijing, China; ^3^ National Institute for Genomics and Advanced Biotechnology (NIGAB), Islamabad, Pakistan; ^4^ Nuclear Institute for Agriculture and Biology College, Pakistan Institute of Engineering and Applied Sciences (NIAB-C, PIEAS), Faisalabad, Pakistan; ^5^ Department of Botany and Plant Sciences, University of California, Riverside, Riverside, CA, United States

**Keywords:** gamma rays, bio fortification, mineral deficiency, *Oryza sativa*, phytic acid

## Abstract

Low phytate level is a desirable trait because it promotes mineral bioavailability and thus offers a solution to tackle mineral deficiencies. The objectives of the present study were to characterize low phytate (lpa) Basmati rice mutants for the identification of novel mutations in target gene(s) and to develop a PCR-based CAPS (cleaved amplified polymorphic sequence) marker for low phytate Basmati rice. For this purpose, cultivar Super Basmati (Q4) was irradiated with gamma rays (^60^Co source) and three mutants named Q1 (lpa-5-9), Q2 (lpa-9-13), and Q3 (lpa-59-14) were isolated. Four genes previously been reported for the low phytic acid trait in rice were sequenced in these mutants and no mutation was observed in Q1 and Q2. However, in Q3 (lpa14) mutant a novel mutation in *OsMRP5* gene (*LOC_Os03g04920`*) was detected. Sequence analysis displayed a substitution in the first exon of *OsMRP5* at position 1142 bp resulting in the amino acid change from glycine (Gly) to alanine (Ala) at position 381a.a. To facilitate low-phytate breeding program, CAPS marker was developed to confirm this mutation site using the restriction digestion by *Alu*I restriction enzyme. After enzyme digestion, Q3 produces four bands (32, 220, 154, and 32 bp) while Q4 (parent cultivar Super Basmati) produces only 3 bands (32, 374, and 32 bp). These results showed that this CAPS marker is 100% linked with this mutation and can be used for future breeding programs. Present findings provided insights in molecular basis of low phytate trait in rice paving the way for developing low-phytate rice varieties through marker-assisted breeding.

## 1 Introduction

In cereals, the major form of phosphorus (P) is myo-inositol-1, 2, 3, 4, 5, 6-hexakisphosphate. This was the first P-containing compound found in seeds and hence known as phytic acid. In humans, due to chelation ability, it is known as an anti-nutrient because it reduces the bioavailability of minerals i.e., Zn^2+^ and Fe^2+^ (Abid et al., 2017; Akhter et al., 2012; Ibrahim et al., 2022). This prevents the absorption of micro-nutrients and leads to malnutrition. Moreover, in monogastric animals, the digestibility of phytate is less and it is excreted as such from the body which causes environmental pollution (Abdel-Megeed and Tahir, 2015; Cosgrove and Irving, 1980; Kumar et al., 2010; Perera et al., 2018; Pramitha et al., 2021).

Rice (*Oryza sativa)* is a staple food for nearly half of the human population and is also rich in magnesium, phosphorus, manganese, selenium, iron, folic acid, thiamin, and niacin (Ravichanthiran et al., 2018; Ahmad et al., 2022). In terms of global production, it is grown in more than 100 countries with Asia accounting for 90% of the total production (Fukagawa and Ziska, 2019). In Pakistan, it is the 2^nd^ most-produced cereal in the country with 7.2 million metric tons (MMT) of production, a 0.6% share in Pakistan’s GDP, and a US$ 2 billion export value (Anonymous, 2019). Almost 10% of Pakistan’s cultivated land area is covered by this crop (Shakoor et al., 2015). Phytic acid (also known as inositol hexaphosphate and IP6) is the storage compound of phosphorus in crops like rice, maize, barley, wheat, etc (Tong et al., 2017). It is an anti-nutritional factor in the seeds of cereals and legumes (Ibrahim et al., 2022). It is a negatively charged molecule that binds positively charged mineral elements, such as Ca, Mg, P, Mn, and Zn; the process is known as chelation (Jacob et al., 2024). These chelates hamper the bioavailability of mineral elements in the body of non-ruminants; the situation is termed hidden hunger or mineral malnutrition which leads to mineral deficiencies induced physiological disorders such as anemia and osteoporosis (Tan et al., 2013). Developed countries of the world have evolved strategies like food fortification, medication, and food supplementation to combat the above-mentioned physiological disorders; however, these approaches are not affordable in developing countries of the world due to high costs. For developing countries, bio-fortification i.e., enhanced bioavailability of the mineral elements, through genetic modification for low phytate crops, is the most economical and sustainable solution.

Eutrophication of lakes and rivers is another drawback of high phytate crops (Gupta et al., 2015; Dwivedi and Ortiz, 2021). It results in poor bioavailability of micronutrients in the body of ruminants and non-ruminants resulting in mineral malnutrition. It is a proven fact that an increase in inorganic phosphorus is inversely proportional to phytic acid phosphates and testing for an increased inorganic phosphate as compared with phytic acid is a simple, inexpensive, and fast approach to select the low phytate genotypes in various cereals and grains (Cichy and Raboy, 2009). Low phytate/biofortified genotypes/cultivars have been discovered and isolated in rice (*Oryzae sativa* L.; Larson et al., 2000; Qamar et al., 2019; Kumar et al., 2021; Utami et al., 2024), wheat (*Triticum aestivum* L.; Guttieri et al., 2004; Frittelli et al., 2023), soybean (*Glycine max L*.; Hitz et al., 2002; Lin et al., 2024), barley (*Hordeum vulgare* L.; Larson et al., 1998; Rasmussen and Hatzack, 1998; Huo et al., 2023), maize (Raboy and Gerbasi, 1996; Raboy et al., 2000; Liu et al., 2007; Yathish et al., 2023), and pea cultivars (*Pisum sativum* L.; Chigwedere et al., 2023; Subasi et al., 2024; Warkentin et al., 2012).

Low phytate mutants have enhanced bioavailability of mineral elements as compared to wild type parental genotypes (Pramitha et al., 2021). Developed countries of the world have evolved strategies like food fortification, medication, and food supplementation to combat mineral deficiencies-induced physiological disorders like iron deficiency-induced anemia during pregnancy, osteoporosis, etc. However, these approaches are not affordable in developing countries of the world due to high costs (Qamar et al., 2019; Perera et al., 2018). As rice is the staple food for the majority of the world population, the aim of producing low phytate mutants rice has great significance to enhance the bioavailability of essential micronutrients (Tan et al., 2013). The development of low-phytate maize, rice, soybean, wheat, and barley by United States Department of Agriculture are important examples to address mineral deficiencies through bio-fortification intervention (Raboy, 2002; Dorsch et al., 2003). Numerous genes that may be responsible for the metabolism of inositol phosphates and PA synthesis have been recognized in rice including, 2-phosphoglycerate kinase (2-PGK), OsMRP5, OsSULTR3;3, inositol monophosphates (IMP), inositol-pentakisphosphate 2-kinase 1 (IPK1), 1D–Myo-inositol 3-phosphate synthase (MIPS), inositol polyphosphate 2-kinase 2 (IPK2), inositol 1,3,4-trisphosphate 5/6-kinase (ITP5/6 K) and myoinositol kinase (MIK) (Suzuki et al., 2007; Kim et al., 2008a, b; Zhao et al., 2016; Li et al., 2014).

Despite highly efficient, fast, and low-cost colorimetric assays that are readily available, the DNA markers such as cleaved amplified polymorphic sequences (CAPS) are being used for the molecular characterization of crops in the world’s major breeding programs (Thiel et al., 2003; Varshney, 2009; Venegas et al., 2022). DNA markers have become a substitute for labor-intensive phenotypic assays (Thiel et al., 2003). CAPS markers are used because these are efficient in detecting polymorphism and are co-dominant with simple interpretation. Marker-assisted selection has been made easier using CAPS markers (Alavi et al., 2009; Song et al., 2023). The CAP1/Hinc II and CAP3/Hpy 99I are recommended and will be useful for the improvement of blast resistance, especially for the early-season indica rice (Lestari and Koh, 2013; Wang et al., 2012; Fan et al., 2023). Four markers have been derived from mutations in four genes that will be useful for enhancing/regulating SPC in pigeon pea crop improvement (Obala et al., 2019). In wheat, various CAPS markers have been developed for stripe rust resistance gene (Rani et al., 2019), leaf rust resistance gene, Lr-1 (Liu et al., 2022a) and yield related QTLs (Duan et al., 2020). In barley, CAPS markers were identified for the spot blotch susceptibility gene (Leng et al., 2018, 2020) and leaf rust resistance gene Rph28 (Mehnaz et al., 2021). Similarly, in maize, CAPS markers have been developed for cytoplasmic male sterility restorer gene (Liu et al., 2022b), drought resistant genes (Liu et al., 2015) and using SNPs for sugarcane mosaic virus resistance gene (Quint et al., 2002).

Through induced mutagenisis several mutants has been developed with number of useful mutations for biotic and abiotic stress tolerance in coarse and Basmati rice (Hameed et al., 2023; Ashraf et al., 2021; Amjad et al., 2022; Zafar et al., 2020; Qamar et al., 2012; Ashraf et al., 2007a, b; Qamar et al., 2005; Cheema et al., 2004; Ashraf et al., 2003; Rashid et al., 2003; Ashraf et al., 2021; Mumtaz et al., 2020, 2018). For the development of novel traits, induced mutation is a biologically safe and non-transgenic approach (Qamar et al., 2024; Rizwan et al., 2019). In our previous reports regarding development of low phytate rice mutants, three homozygous lpa mutants lpa-5-9, lpa-9-13, and lpa-59-14 were developed through induced mutations (gamma rays ^60^Co) and identified by colorimetric and High-performance Liquid Chromatography (HPLC) analysis (Cheema et al., 2008; Qamar et al., 2019). In these mutants, phytic acid content reduced to 54-63% but with poor germination and yield. Previously reported Lpa1-CAPS and Lpa1-InDel and functional molecular markers (Tan et al., 2013) were applied but indicated the absence of the Z9B-Lpa allele and XS-Lpa mutation in the *OsMRP5* gene in these mutants (Qamar et al., 2019). Based on these previous studies, we proposed that there must be new mutations or novel alleles in our developed low phytate mutants that needs to be identified. Further attempts were made to improve germination and yield traits through hybridization, and backcross breeding.

In this view, present study was conducted for molecular characterization of low phytate basmati mutants with improved germination. Efforts were made to characterize low phytate (lpa) Basmati rice mutants for the identification of novel mutations in target gene(s) and to develop a PCR-based CAPS (cleaved amplified polymorphic sequence) marker for new mutation in low phytate Basmati rice. In this study, a CAPS marker for a new mutation in *OsMRP5* gene in low phytate Basmati rice mutant was developed.

## 2 Materials and methods

### 2.1 Experimental material

The experimental material consisted of three low phytate mutant lines Q1 (lpa-5-9), Q2 (lpa-9-13), and Q3 (lpa-59-14) and non-low phytate Super Basmati with optimum germination and yield. These low phytate mutants (~58% reduction in phytic acid) were developed using gamma radiations (^60^Co source) at 250Gy dose under an IAEA project RAS7/014. Other materials include different breeding populations developed through the hybridization of LPA mutant lines with Super Basmati and backcross breeding.

### 2.2 Confirmation of lpa mutants using a colorimetric assay

Colorimetric Assay was performed according to the method previously described by (Chen et al., 1956; Qamar et al., 2019). Briefly, healthy seeds of each genotype were collected and the husk was removed by hand separately. Seeds were cut with the help of a sharp blade into two parts. One part containing the embryo was stored for germination and another half part was grounded. Grinding was performed by using a machine “Geno-Grinder”, for this purpose, half seed was kept in a 2mL Eppendorf tube (EP) and one metal ball was added into the EP tube. EP tubes were dipped in liquid nitrogen for one minute. The machine’s speed was set at 1450 rpm for 40 seconds. After grinding into a fine powder, 200 µL of 0.4 M HCl was added to each sample. Sample tubes were shacked gently by the hand and kept at 4°C for overnight (incubation). The next morning, 10 µL of the extracted sample was mixed with 90 µL of ddH_2_O, and 100 µL of colorimetric reagent was added. Incubation at room temperature for 1 hour was performed.

#### 2.2.1 Colorimetric reagent

Mix 1 volume (1 mL) of 6 N sulfuric acid (Dilute 18 mL of commercial-grade sulfuric acid to 108 mL of ddH_2_O. For this purpose, 90 mL of ddH_2_O was taken and 18 mL of H_2_SO_4_ was added slowly) with 2 volumes (2 mL) of ddH_2_O and 1 volume (1 mL) of 2.5% ammonium molybdate (2.5 g of ammonium molybdate (NH_4_)_6_ Mo_7_O_24_.4H_2_O was dissolved in 70 mL ddH_2_O and adjusted the final volume up to 100 mL), then add 1 volume (1 mL) of 10% ascorbic acid (10 g of ascorbic acid was dissolved in ddH_2_O and the final volume was adjusted up to 100 mL). The solution was stored at 2-4°C for use. It can be stored for up to 7 weeks but better to prepare fewer amounts and mix well. On each day it was prepared freshly. All these operations were carried out in the laminar air hood and special care was taken during handling with acids.

#### 2.3 Germination test

For further confirmation of low phytate mutants, we sow the already screened seeds from colorimetric assay on a half-strength MS medium in glass bottles (diameter = 6 cm, and 18 cm in height). These bottles were placed in the growth chamber at 23-26 °C for 16 hours of light and 8 hours of dark, while the relative humidity was kept at 70%. Subsequently, these seeds were confirmed through genotyping (sequencing).

### 2.4 Molecular characterization of low phytic mutants for reported mutations in genes

In present study, three low phytate (lpa) mutants named as Q1 (lpa-5-9), Q2 (lpa-9-13), and Q3 (lpa-59-14), along with parent cultivar Q4 (Super Basmati) were used for different evaluations. Four low phytic acid mutant genes (**Table 1**) were selected to find any mutation in our lpa mutants. Primers for these selected genes were designed (**Table 2**). Some walking primers according to the PCR product size were also designed for complete sequencing. For this purpose, 8 days old seedlings were used for DNA isolation following the CTAB method. After PCR amplification, the PCR product was sent to BGI Genomics, Beijing Company for sequencing.

**Table 1:** The four reported low phytic acid mutant genes used for sequencing.

Gene code	Gene name	Locus. I. D
LPA1	2-phosphoglcerate kinase (*2-PGK*)	*LOC_*Os02g57400
LPAN15-186	Myo-inositol kinase (*OsMIK*)	*LOC_*Os03g52760
LPA, XS 110-2, XS-LPA2	*OsMRP5*	*LOC_*Os03g04920
MH86-LPA	*OsSULTR3;3*	*LOC_*Os04g55800

**Table 2:** List of primers used for amplification of lpa genes.

Primer name	Primer Sequences (5´-3´)	PCR Size (bp)	Reference
*LOC_*Os02g57400-F	GGGTATCACGACAGTAGTTTC	2162	Zhao et al., 2008
*LOC_*Os02g57400-R	ACCGAGTTCAGAGCACATCTG		
*LOC_*Os03g52760-F	CTCCATTCCACGCGATGCCGT	2771	Kim et al., 2008b
*LOC_*Os03g52760-R	GTGTGGAAACTTGGATGTCAAG		
L2W1F	GGTTGGAGTACCAACCTTCGATC		
L2W1R	GACAAGAGCTACCTGAAGTTCTG		
*LOC_*Os03g04920-F	CTTTCTCGCTCGACGAGGAGGT	6025	Xu et al., 2009
*LOC_*Os03g04920-R	GCTCATATACACCTCGGCTTGTG		
L3W2F	CGGCCATGGTGTACAGGAAGGG		
L3W3F	CCTTAGTGTGGTGAGGGGTATG		
L3W4F	GAGACTATGCATGATCATAGGG		
L3W5F	GATACTACACCATCTGGTCGA		
*LOC_*Os04g55800-F	CCTAGCTCTCCTTGCAACAAC	5401	Zhao et al., 2016
*LOC_*Os04g55800-R	GGGAAATACGTTGCTGGAGGC		
L4W2F	GGCATGTGGTACGTATCACATC		
L4W3F	GAACCTTGCAACCATGCTGATAG		
L4W4F	GGAAGAACTGAACAGATCTTGC		

### 2.5 Development of CAPS marker to detect the Q3 (lpa-59-14) mutant site in the *OsMRP5*

For the confirmation of the mutation site, we designed a CAPS (cleaved amplified polymorphic sequence) marker (**Table 3**). For this purpose, we performed a PCR, and then the PCR product was digested with the *Alu*I (AGCT) restriction enzyme. 1 µl of *Alu*I with 3 µl of cut-smart buffer was added to 30 µl of PCR product. This mixture was kept at 37°C for 90 minutes. Then it was run on the 1% agarose gel. After the electrophoresis, the size of the band was measured under UV light using Gel Doc Quantity One software (Version 4.5.1, BIO-RAD^®^).

**Table 3:** PCR primers used for the MRP5 CAPS markers.

Primer name	Sequence (5´-3´)
MRP5CAPSF	ATTGTCTCCTATGTTGGCCC
MRP5CAPSR	AGGAACTGAGGCAGCAATTG

### 2.6 Multiple sequence alignment and phylogenetic analysis

The amino acid sequences of MRP5 and their orthologue genes from different species such as rice, maize, sorghum, and Arabidopsis were retrieved from Ensembl plants (https://plants.ensembl.org/index.html). The BioEdit tool was used to align the sequences by using ClustalW multiple alignments. MEGA7 software was utilized for the construction of a phylogenetic tree. For this purpose, a neighbor-joining method with 1000 bootstraps was used.

## 3 Results and discussion

### 3.1 Confirmation of the *lpa* mutants through colorimetric assay

**Figure 1:**
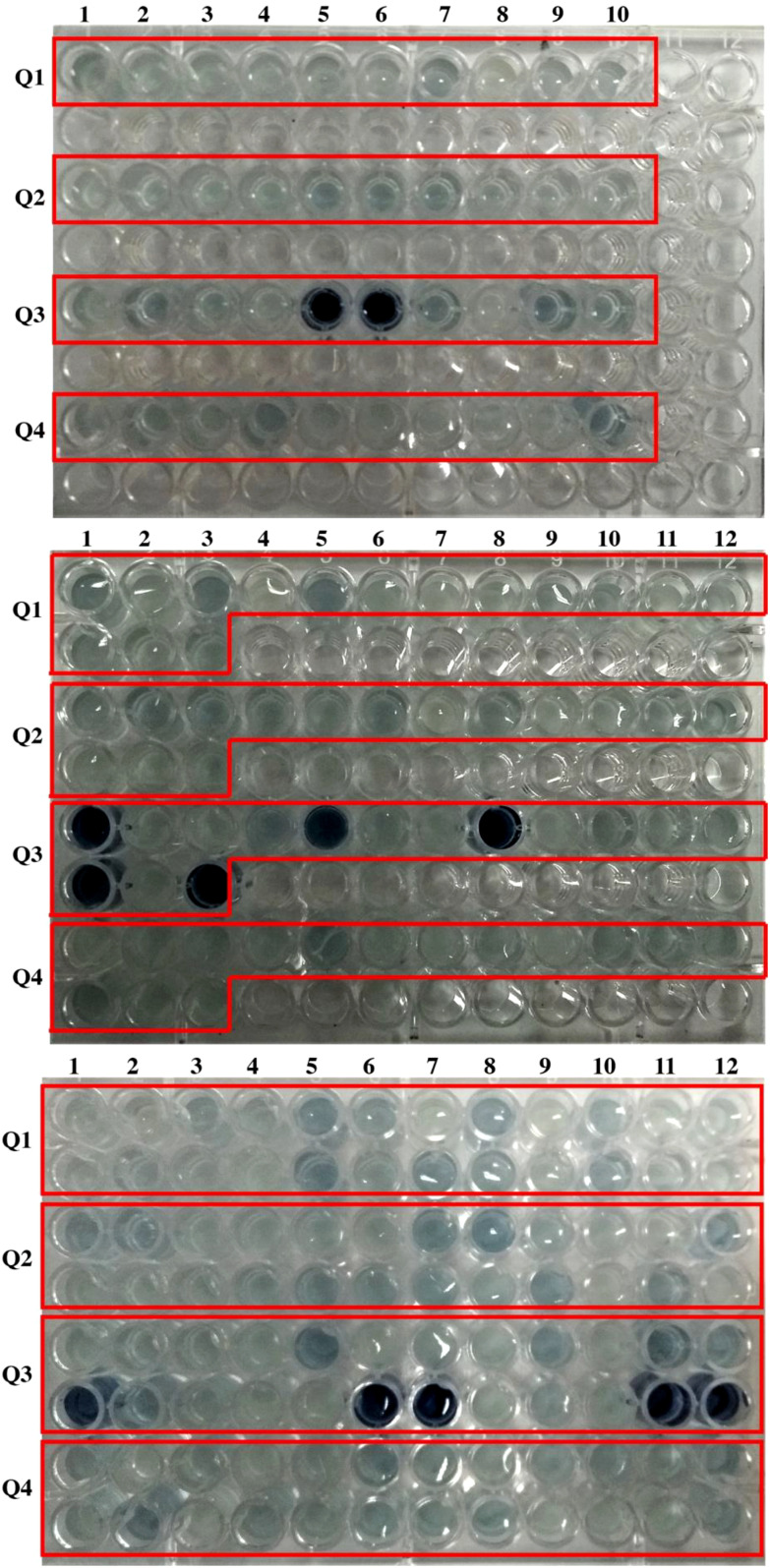
Colorimetric assay for the detection of low phytate mutants.

Colorimetric assays are a standard approach for detecting and quantifying phytate levels in plant samples. Plant types that have been genetically engineered or chosen to generate seeds or grains with lower amounts of phytate are known as low-phytate mutants. Plant breeders and farmers are interested in developing low phytate mutants because phytate may bind to key minerals such as iron, zinc, and calcium, rendering them less accessible for absorption by the human body. In these tests, a reagent is added to a plant sample, which interacts with inorganic P to cause a color change. Decreases in seed phytate increase seed inorganic P (the “High Inorganic P” phenotype) and since there is low inorganic P in wild-type seeds, this increase is easy to detect with this colorimetric assay. During this study, 49 seeds of each genotype (Q1-Q4) were used for colorimetric assay (**Figure 1**). Individual seed extracts were visually observed. There was no detection of blue color in the Q1 and Q2 genotypes. While in the Q3 genotype, 12 out of 49 seeds showed dark blue color which indicates 3:1 segregation ratio (**Figure 1**).

### 3.2 Confirmation of lpa through germination

**Figure 2:**
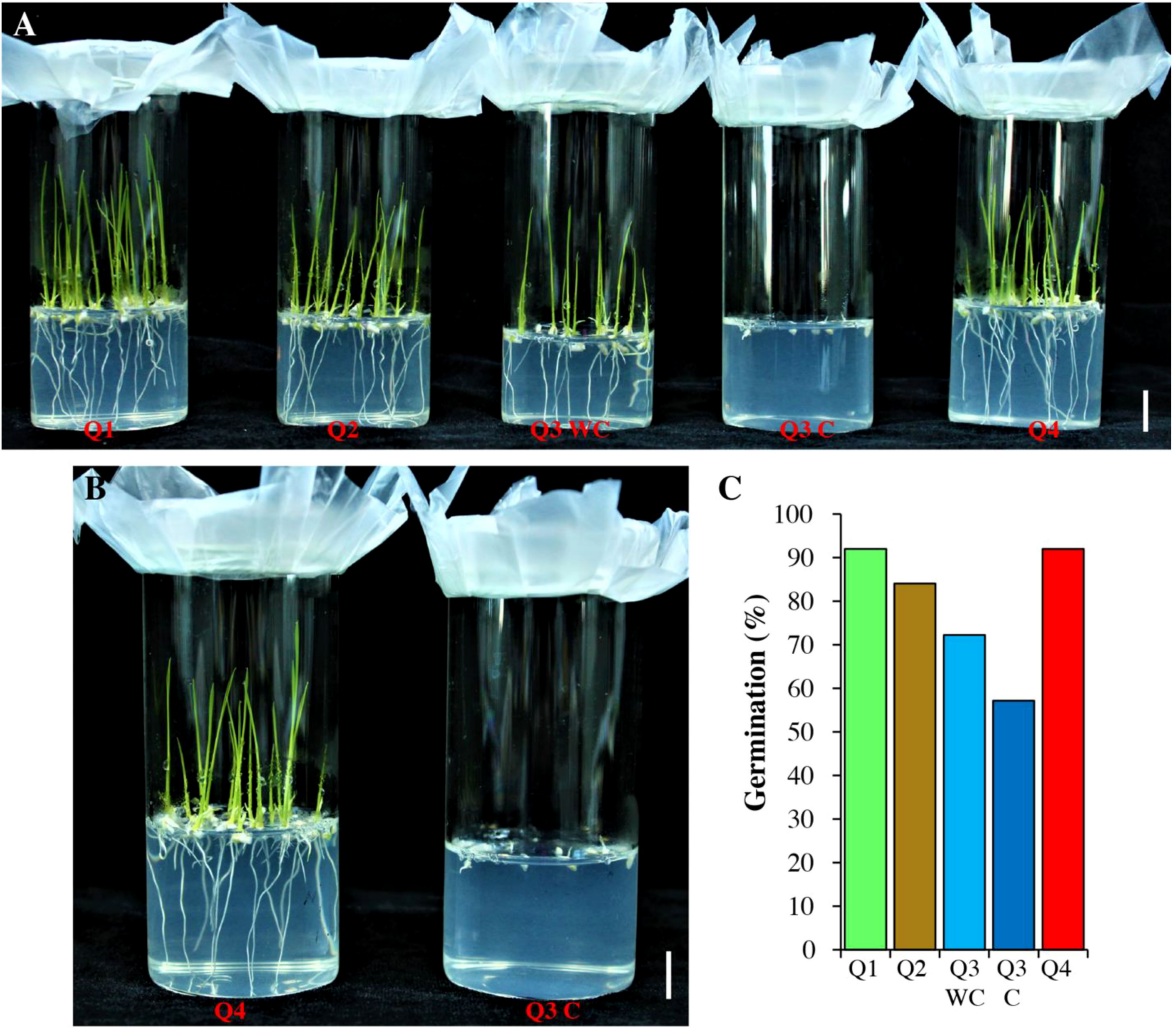
Germination test of low phytate genotypes. **(A)** Four genotypes Q1-Q4 were grown on ½ MS medium. Pictures were captured 5 days after sowing. WC, without color; C, colored. **(B)** Comparison of Q3 C with Q4. Scale bar = 1 cm. **(C)** Quantification of germination (%).

Previously it is reported that low phytate mutants have poor germination (Zhao et al., 2008; Qamar et al., 2019). In the current study, we also checked the germination of pre-selected seeds (**Figure 2**; **Table 4**). The Q1, Q2 and Q4 genotypes showed similar germination 92%, 92% and 84%, respectively. Seeds from Q3 were divided into two parts (blue color Q3C and without color Q3 WC) and sown separately. The germination rate of seeds showing blue color was lower as compared with Q3 WC and Q4. The seedling growth rate of Q3C is also very slow as compared with Q4 (**Figure 2B**). This indicates that these are the possible true low phytate mutant seeds.

**Table 4:** Germination test for the screened seeds.

Sr. No.	Genotypes	No. of plants screened	No. of germinated plants
1	Q1	25	23
2	Q2	25	21
3	Q3 (color)	7	4
	Q3 (without color)	18	13
4	Q4	25	23

### 3.3 Genotyping of low phytate mutants

Twelve seeds (already screened through colorimetric assay) of each genotype (Q1-Q4) were used for genotyping (**Table 5**). Q1 and Q2 both showed WT (9/12, AA) and heterozygous (3/12, Aa) plants, while Q4 have all (12/12, AA) WT plants. We also used 9 plants of Q3 WC (no color in colorimetric assay), all the plants in this genotype were also WT (AA). These results further support the findings (seeds are not pure) of the colorimetric assay and germination test.

**Table 5:** Sequencing results of low phytate mutants.

	AA	Aa	aa
Number of Plants	**Q1 =** 1,2,3,4,5,6,8,9,10	7,11,12	
	**Q2 =** 13,14,17,18,19,20,21,22,24	15,16,23	
	**Q4 =** 25,26,27,28,29,30,31,32,33,34,35,36		
	**Q3CL =** 37,38,39,40,42,43,44,45 41-no sequencing		

### 3.4 Identification of mutation sites in the lpa-59-14 mutant

**Figure 3:**
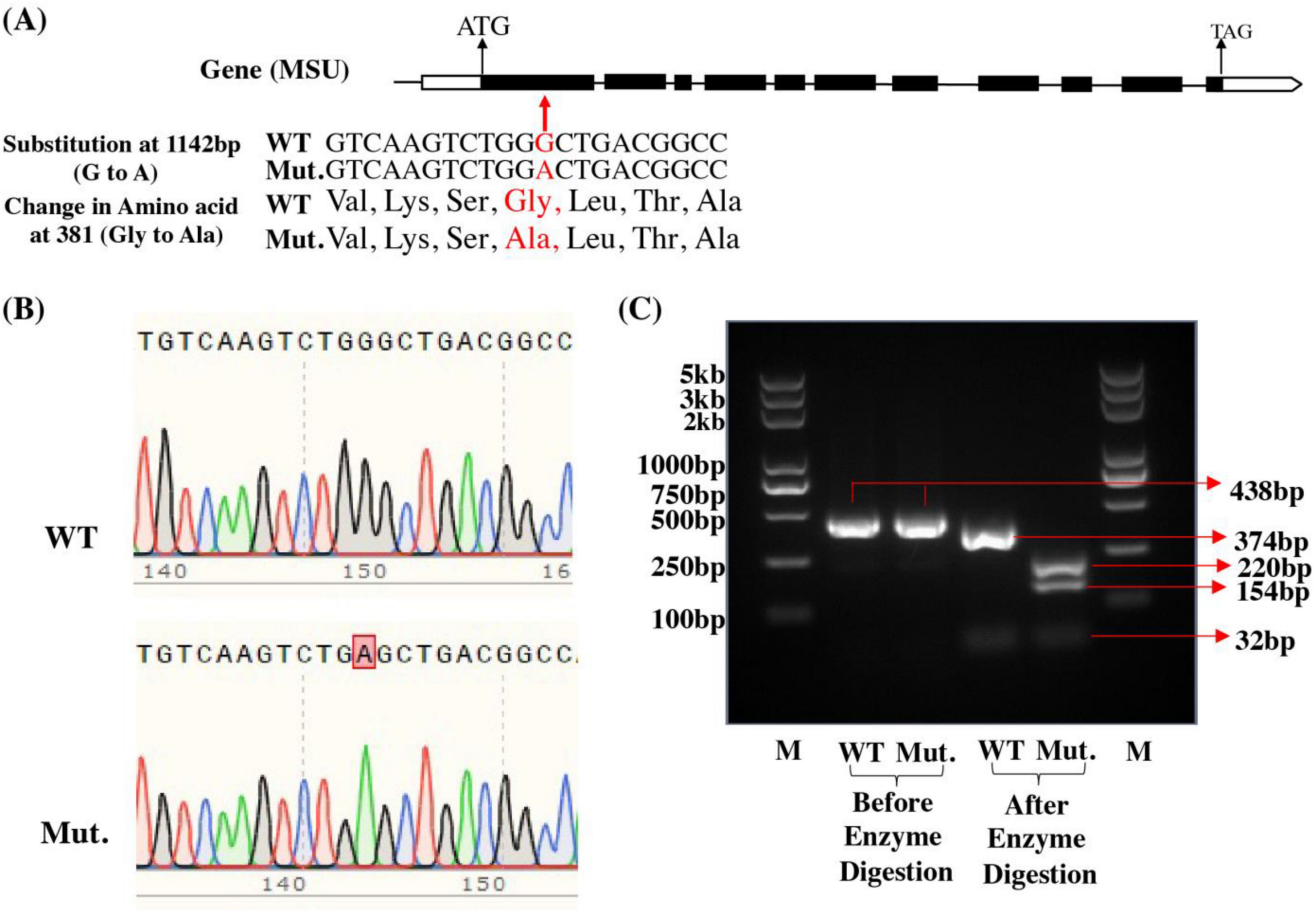
Confirmation of LPA3 gene. **(A)** Gene structure and mutation site. **(B)** Sequencing results of WT and mutant (Mut). **(C)** Confirmation of mutation through Alu1 enzyme digestion. PCR product size is 432bp. After digestion with the restriction enzyme *Alu*I (recognition site: AGCT), WT(Qamar4) produced 3 bands: 32bp, 374bp, 32bp; the lpa-59-14 mutant (Qamar3) produced 4 bands: 32, 154, 220, 32bp.

The results of sequencing of the PCR product revealed that we did not find any mutation sites in Q1 and Q2 genotypes in the four reported low phytate mutant genes in rice (**Table 1**). However, we did find a mutation site in Q3 (lpa-59-14) in the *OsMRP5* gene (*LOC_Os03g04920*). There is a substitution in the first exon at position 1142 bp (G to A). Due to this substitution, the amino acid sequence also changed from Gly to Ala at position 381a.a (**Figure 3A**). This was also confirmed through sequencing (**Figure 3B**). There was a single peak in the mutant which further confirmed the G to A substitution.

### 3.5 Use of a CAPS marker to detect the lpa-59-14 mutant site in the *OsMRP5*

To further confirm this mutation site, we designed a CAPS (cleaved amplified polymorphic sequence) marker. For this purpose, we performed a PCR, and then the PCR product was digested with the *Alu*I restriction enzyme. After enzyme digestion, Q3 produces 4 bands (32, 220, 154, and 32 bp) while Q4 produces only 3 (32, 374, and 32 bp) bands (**Figure 3C**). This marker is 100% linked with this mutation and can be used for future breeding programs. Previously, CAPS markers for low phytate grain trait were developed for maize kernels (Abhijith et al., 2020) and Rice (Tan et al., 2013) for different genes but this is the first study for the development of functional molecular marker based on *MRP5* gene which adds further diversity into our pool of molecular markers.

### 3.6 Multiple sequences alignment and phylogenetic analysis

**Figure 4:**
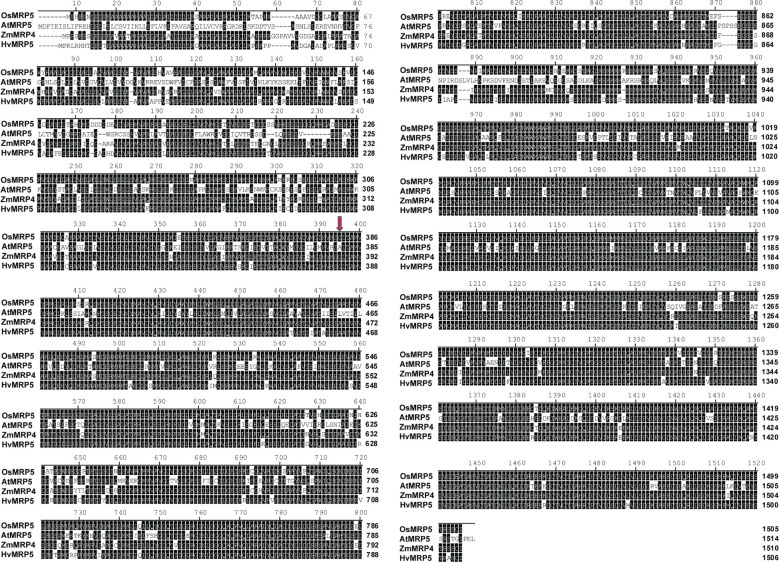
Amino acid sequences alignment of MRP5 (Red arrow represents the mutation site).

**Figure 5:**
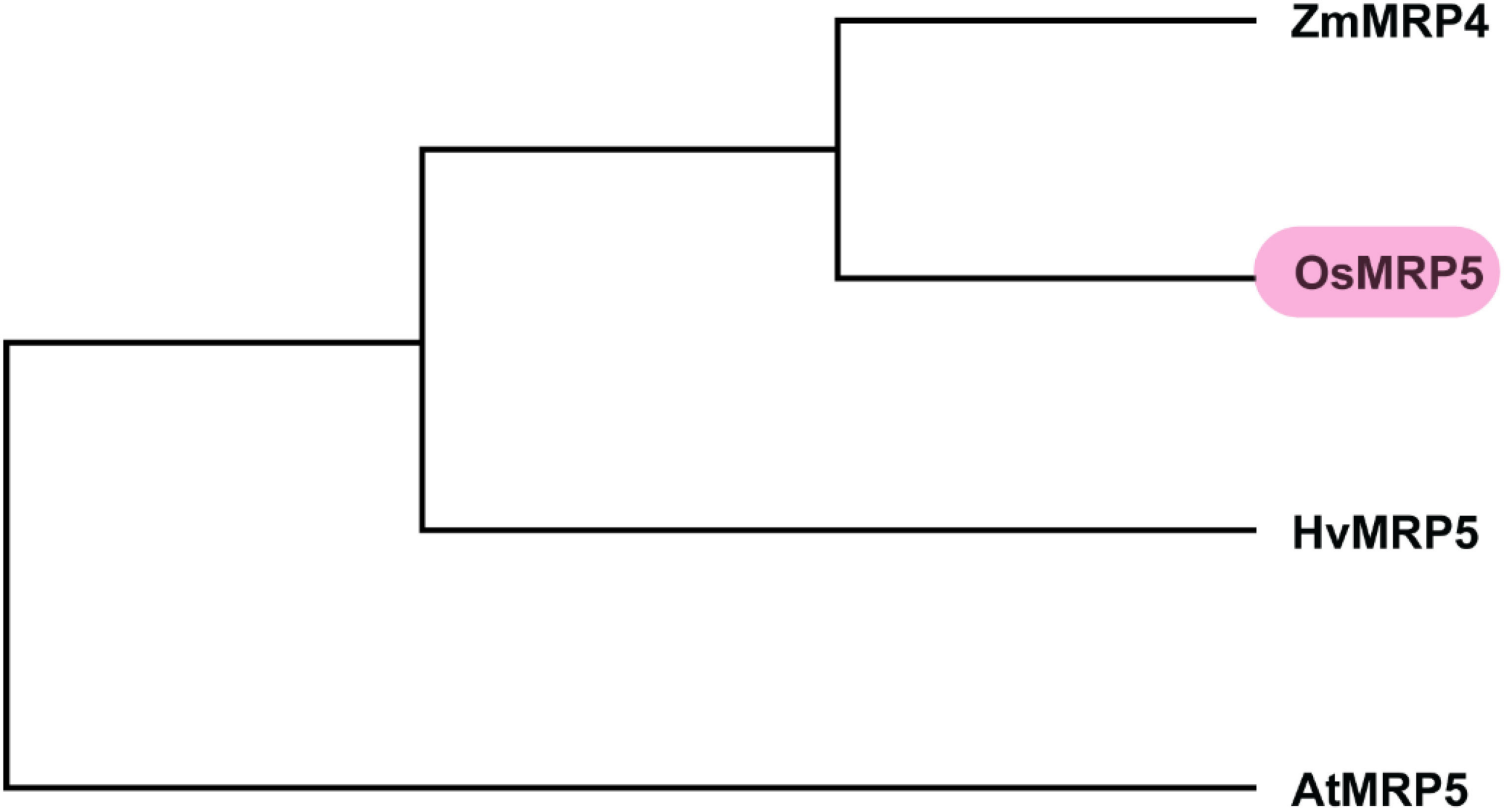
Phylogenetic analysis of MRP5.

To scan the sequence of *MRP5* gene across different cereals versus model plant Arabidopsis, we did a sequence alignment of amino acid sequences of MRP5 (**Figure 4**). A phylogenetic tree (**Figure 5**) was constructed using orthologue genes from different species such as rice, maize, barley, and Arabidopsis. Results indicated that all these genes are closely related to each other’ but OsMRP5 is more closely related to maize MRP4 which suggest their more recent evolution from a common ancestor.

## 4 Conclusion

In conclusion, we were able to detect a novel mutation in *OsMRP5* gene (*LOC_ Os03g04920*) in Q3 (lpa-59-14) mutant. Sequence analysis displayed a substitution in the first exon of *OsMRP5* at position 1142 bp resulting in the amino acid change from glycine (Gly) to alanine (Ala) at position 381a.a.a. A breeder friendly CAPS marker for a new mutation in OsMRP5 gene in low phytate Basmati rice mutant was developed. This CAPS marker provided 100% confirmation of new low phytic acid mutation and can be used for future marker assisted breeding programs.

## Data Availability

The raw data supporting the conclusions of this article will be made available by the authors, without undue reservation.
